# Effects of maternal temperament on uterine and umbilical blood flow, expression of angiogenic proteins, and fetal growth in pregnant ossimi sheep

**DOI:** 10.1590/1984-3143-AR2024-0125

**Published:** 2025-07-07

**Authors:** Mohammed Elmetwally, Fatma Adlan, Basma Hendam, Alaa Samy, Shaymaa Rezk, Samah Lasheen, Heba Mohram, Sara Shalapy, Reham Fahmy, Amy Jo Montgomery, Yasser Y Lenis, Amira Mostagir

**Affiliations:** 1 Department of Theriogenology, Faculty of Veterinary Medicine, Mansoura University, Mansoura, Egypt; 2 Center for Reproductive Biotechnology Faculty of Veterinary Medicine, Mansoura University, Mansoura, Egypt; 3 Mansoura Veterinary Teaching Hospital, Mansoura University, Mansoura, Egypt; 4 Department of Animal Wealth Development, Faculty of Veterinary Medicine, Mansoura University, Mansoura, Egypt; 5 Department of Surgery, Anesthesiology, and Radiology, Faculty of Veterinary Medicine, Mansoura University, Mansoura, Egypt; 6 Department of Cytology and Histology, Faculty of Veterinary Medicine, Mansoura University, Mansoura, Egypt; 7 Department of Internal Medicine and Infectious Diseases, Faculty of Veterinary Medicine, Mansoura University, Mansoura, Egypt; 8 Oncology Center, Faculty of Medicine; Mansoura University, Mansoura, Egypt; 9 School of Medicine, St. George’s University, West Indies, Grenada; 10 Research Group OHVRI, School of Veterinary Medicine, Faculty of Agrarian Science, Universidad de Antioquia, Medellín, Colombia

**Keywords:** sheep, temperament, color doppler, fetal heart rate, pregnancy, arena test

## Abstract

This study aimed to examine the efficacy of Doppler and B-mode sonography in evaluating the impact of maternal temperament on hemodynamic alterations in both the fetus and uterus during ovine gestation in addition to alteration of the angiogenic protein expression and fetal biometry. Twenty Ossimi ewes were divided into two groups, more reactive (MR: 12 sheep) and less reactive (LR: 8 sheep). Several endpoints were assessed every 2 weeks (wk) from breeding to wk 20 of pregnancy. Blood samples were taken to evaluate the expression of angiogenic proteins at parturition. The resistance (RI) and pulsatility (PI) indexes of the uterine (UtA) and fetal umbilical arteries (UMA) were measured. Biometry includes: diameter of amniotic vesicles (AVD), umbilicus (UMD), fetal thoracic diameter (FTD) and metacarpal length (MCL). The UtA-PI was higher in MR compared to LR ewes between 6-12 wks of gestation (P>0.01), while a tendency was recognized at wk 14 (P=0.054). The same was true for UtA-RI during the first 8 wk of pregnancy (P < 0.03) when MR and LR animals were compared. Similarly, UMA-RI was higher in fetuses of MR than LR ewes at wk 14 (P<0.0003) and 20 (P<0.02) of pregnancy. The differences in UMA-PI reached significance at wk 6, 8, 10, and 20 (P<0.0 –0.003). Furthermore, significant changes in fetal biometry were investigated. The expressions of *VEGF, NOS3* and *HIF 1α* were increased in the less reactive sheep (P<0.001). In conclusion, the maternal temperaments affect the Doppler, B mode as well as the expressions of mRNAs for *VEGF, NOS3* and *HIF 1α* genes at time of parturition.

## Introduction

Stress is a reflex reaction that can have a variety of detrimental effects and arises when an animal is unable to adjust or deal with the negative impacts of numerous situations (Elmetwally et al., 2021). Stress-inducing stimuli don't always hurt, but psychological states like dread or worry cause the body to react physiologically in ways that could harm the animal. An animal's response to a perceived threat can be behavioral, autonomic, endocrine, or immunological in order to preserve homeostasis (Elmetwally et al., 2021). Stress in the post-conceptional period has been shown to cause shorter gestation periods and low birth weight in goats (Elmetwally et al., 2021). Recent human studies have linked low offspring birth weight to prenatal stress, which may contribute to intrauterine foetal growth restriction (Sertie et al., 2020; Su et al., 2021; Thomason et al., 2021).

Although sheep production fluctuates depending on market demand, it is a crucial aspect of animal husbandry that holds significant importance in many nations (He et al., 2023). Because of their docile nature and high degree of environmental adaptability, cultures have long raised sheep for their meat, milk and wool (Amane et al., 2023).

Though research on conditions involving predicted recurrent stress, such as social isolation, transit, or unpleasant handling, has yielded conflicting findings, detailed descriptions of pregnant stress in sheep remain rare (He et al., 2023).

Doppler ultrasonography has been shown to be an accurate non-invasive diagnostic tool for studying uterine and umbilical blood flow changes during pregnancy and puerperium in small ruminants (Elmetwally and Bollwein, 2017; Elmetwally, 2012; Elmetwally et al., 2016a). Season, nutrition, social structure, and stress are all factors that influence reproduction performance in small ruminants (Elmetwally et al., 2016b, 2021; Everett-Hincks and Dodds, 2008; Thiéry and Martin, 1991). In women and goats maternal anxiety has negative adverse influences on uterine artery Doppler parameters, fetal growth, gestational length effects on birth weight outcomes and uterine blood flow (Charrois et al., 2020; Ding et al., 2014; Elmetwally et al., 2021; Helbig et al., 2013). According to Doppler sonography, anxious women had more abnormal variables in uterine artery blood flow than less anxious women (Asnafi and Hajian, 2011; Teixeira et al., 1999).

Uterine artery PI (Konchak et al., 1995) and uteroplacental flow resistance (Teixeira et al., 1999) were higher in stressed pregnant women than in non-stressed pregnant women. Similar findings in goats have recently been published (Elmetwally, 2012; Elmetwally et al., 2021). Prenatal maternal stress and females with more daily stressors and a depressed mood during the first trimester all reduced pregnancy duration, birth weight, and foetal head circumference (Cattane et al., 2021; Dunkel Schetter and Tanner, 2012; The American College of Obstetricians and Gynecologists, 2015). Similarly, babies born to stressed mothers had shorter femurs, as well as smaller abdominal and head circumferences (Finken et al., 2018; Sarkar et al., 2017; Wu et al., 2020).

The circulatory system is the earliest biological system to develop in mammals (Sasaki et al., 2017). There are two primary methods by which vessels form: vasculogenesis and angiogenesis (Semenza, 2007). Vasculogenesis is the process through which the major vascular plexus develops during embryonic development, whereas angiogenesis is the process by which new blood vessels are formed from existing vessels (Carmeliet, 2003). It was identified that changes of the fetus are connected to structural alterations in the placenta as the number of cotyledons and the placental weight are reduce, deviations of the fetus are connected to structural alterations in the placenta (Semenza, 2007). In addition, processes of vasculogenesis and angiogenesis are the foremost processes influential maturation and development of placenta as they directly affecting fetoplacental blood flow in addition to fetal development (Semenza, 2007). During pregnancy, the growth and the importance of placenta focus on the development of its vascular supply and an increase in umbilical and uterine blood flow. Furthermore, vascular endothelial growth factor (VEGF) and nitric oxide synthase (NOS) perform critical functions in expansion of the placenta of sheep (Semenza, 2007). It is noted that the patterns of VEGF and NOS expressions were tangled with the angiogenesis and vasculogenesis processes in the ovine placenta throughout normal pregnancies (Semenza, 2007). Meanwhile, the nuclear transcription factor hypoxia-inducible factor (HIF) might simplify placental oxygen transport via boosting erythropoiesis and placental angiogenesis. Damage of HIF-1α protein is repressed in hypoxia and it gathers in the nucleus to triggers genes through hypoxia response essentials (Semenza, 2007).

Our hypothesis is that maternal temperament may affect the uterine and fetal blood flow indices as well as fetal biometry during pregnancy in sheep. To the best of our knowledge, there is a scarcity of data on the effects of maternal temperament on maternal, fetal, and pregnancy-related ultrasonic parameters in sheep. The current prospective study aimed to perceive if maternal temperament influences Doppler velocimetry variables, potentially leading to altered fetal growth as well as the expression of angiogenic protein genes.

## Methods

### Ethics statement

All used protocols in this study were approved by the Committee on the Ethics of Animal Experiments of the Faculty of Veterinary Medicine, Mansoura University Code No. VM.R.22.12.36. This means that all sheep used did not suffered from any pain, stress and harm during the experimental period. All the collected samples were obtained by an expert veterinarian to ensure that all animals were welfared.

### Animal allocation and experimental design

This study was conducted between October 2023 and April 2024. Animal experiments described in this article were conducted in accordance with the Guiding Principles for the Care and Use of Research Animals Mansoura University (VM.R.22.12.36). Non lactating pluriparous Ossimi female sheep (n=20 and assorted as: more reactive/anxious (MR: 12 sheep) and less reactive/anxious (LR: 8 sheep) were investigated during the period of this study. Each having three previous parities. The average age of the experimental ewes was 5.5 ± 2.4 y old (mean ± SD, range 3-10 y). The body condition score of the experimental animals was 3-4 (Semenza, 2007) and the average body weight was 54 ± 6.4 kg. The animals were housed in a barn with access to outside runs during the winter, but they were maintained on pasture during late fall and early winter. The base diet during the latter period included pellet feed [amount adjusted to the stage of pregnancy according to the NRC recommendation (National Research Council, 2007: Table 1)].

**Table 1:** Feed ingredients and composition Mineral and vitamin premix is composed of phosphorus,magnesium, potassium, sulphur, chlorine, vitamin B complex, selenium, and yeast.

**Feed ingredients (%)**	
Soybean meal	6
Cotton seed cake	16
Wheat bran	24
Yellow corn	51
Ground limestone	1.7
Common salt	0.8
Mineral-vitamin premix	0.5
**Feed composition (%)**	
Crude protein	14
Ether extract	4
Total digestible nutrients	68
Fiber	8
Ash	6

The experimental animals were allocated into two temperamental groups more reactive and less reactive (LR) after Arena test, a behavioral test used on all the sheep according to Elmetwally et al. (2021). Sheep in the present experiment were synchronized with prostaglandin F2_2α_ [PGF_2α_], analog (125 µg cloprostenol, Schering Plough, USA, i.m) and then mated with a proven fertile male during behavioral estrus. B mode ultrasonography examination was performed on the 25^th^-day post-breeding. No fetal loss was detected in this study. Arena tests for experimental sheep allocation were done according to previous literature (Elmetwally et al., 2021).

### Color Doppler sonography examination of uterine arteries

**Figure 1:**
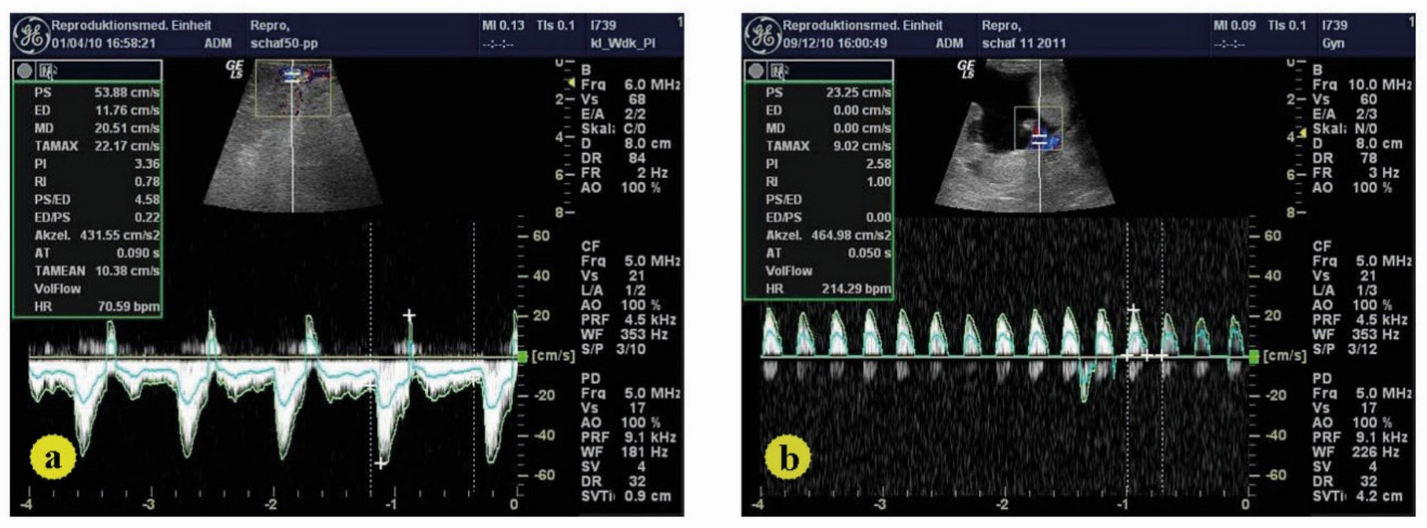
(a) Doppler flow velocity waveforms of uterine arteries in pregnant sheep; (b) Doppler flow velocity waveforms form fetal umbilical artery in pregnant sheep.

Elmetwally et al. (2021) described the use of a LOGIQ 5 Pro ultrasound machine (General Electric’s Healthcare, Solingen) equipped with a linear-array, multifrequency (5-10 MHz) transducer for uterine artery localization and Doppler examination of the pregnant sheep (Elmetwally et al., 2016a). In each uterine artery corresponding to the gravid side, time average maximum velocity (TAMV), pulsatility index (PI) and resistence index (RI) were measured (Figure 1). The Doppler angle was kept between 30 and 40 and the indexes were taken every two weeks from week 2 to week 20 of the pregnancy. The Doppler examination took about 30 minutes for each pregnant sheep. The gestational periods of the experimental sheep that delivered normally and without complications were recorded. The weights of the lambs were taken immediately after birth.

### B-mode sonography

**Figure 2:**
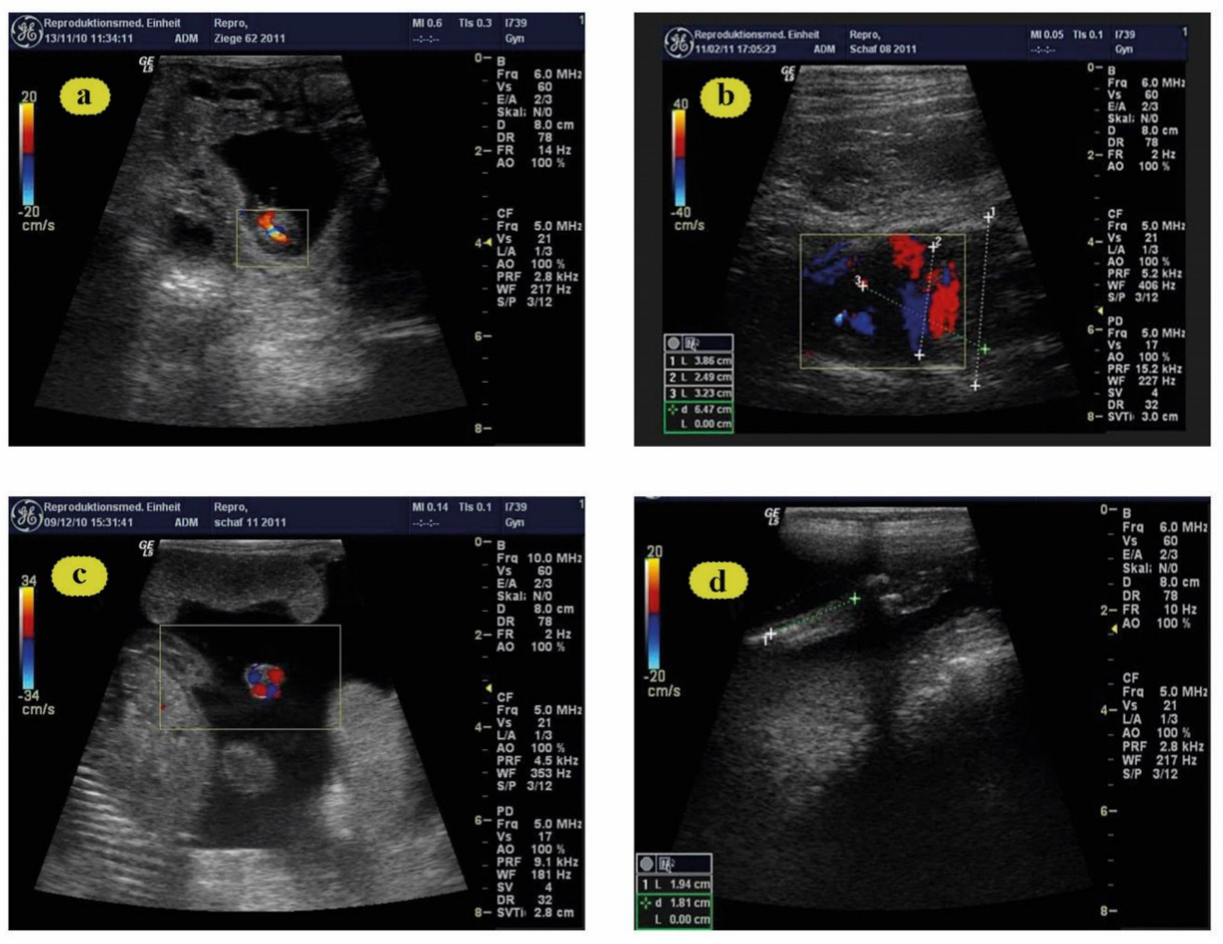
(a) Amniotic vesicle at day 25 of gestation; b: Thoracic diameter at day 100 of gestation; (c) (Wk 14): Fetal umbilical cord diameter at day 110 of gestation (Wk 16) and (d) Fetal metacarpal length at day 56 of gestation (Wk 8).

When possible, the following fetometric endpoints were measured using a 9 MHz linear-array transducer (Sonoscape A5V, Shenzhen, China) from wk 2 after breeding until parturition: diameter of amniotic vesicles (Figure 2a: AVD), fetal thoracic diameter (Figure 2b: FTD), and umbilical cord diameter close to the fetal body (Figure 2c: UMD), metacarpal length (Figure 2d: MCL) according to Elmetwally (2012).

### Tissue sampling and Macroscopic examination

Placental tissue specimens were collected from 5 ewes directly after dropping off the placenta (30 minutes to 21 hours after birth) at the mid portion of the placenta having placentome. Macroscopic inspection was performed on placental samples. Sections of placenta from all sheep were collected as rapidly as possible and stored in 10% buffered formaldehyde for histological analyses.

### Histological analysis

#### Light microscope tissue fixation and processing

All the collected tissue for light microscopy was perfused and fixed with 10% buffered formaldehyde for 72 h, rinsed in phosphate buffered saline (PBS; pH 7.4). Placenta from random locations was trimmed and dehydrated in ascending concentrations of ethyl alcohol, cleared in xylol and impregnated embedded paraffin wax using established methods. Sections of 5μm were collected on glycerol-albumin-coated glass slides and dried for at least 24 h in a 37˚C incubator. Paraffin sections that were kept in the incubator were prepared for staining.

#### Tissue staining

**Figure 3:**
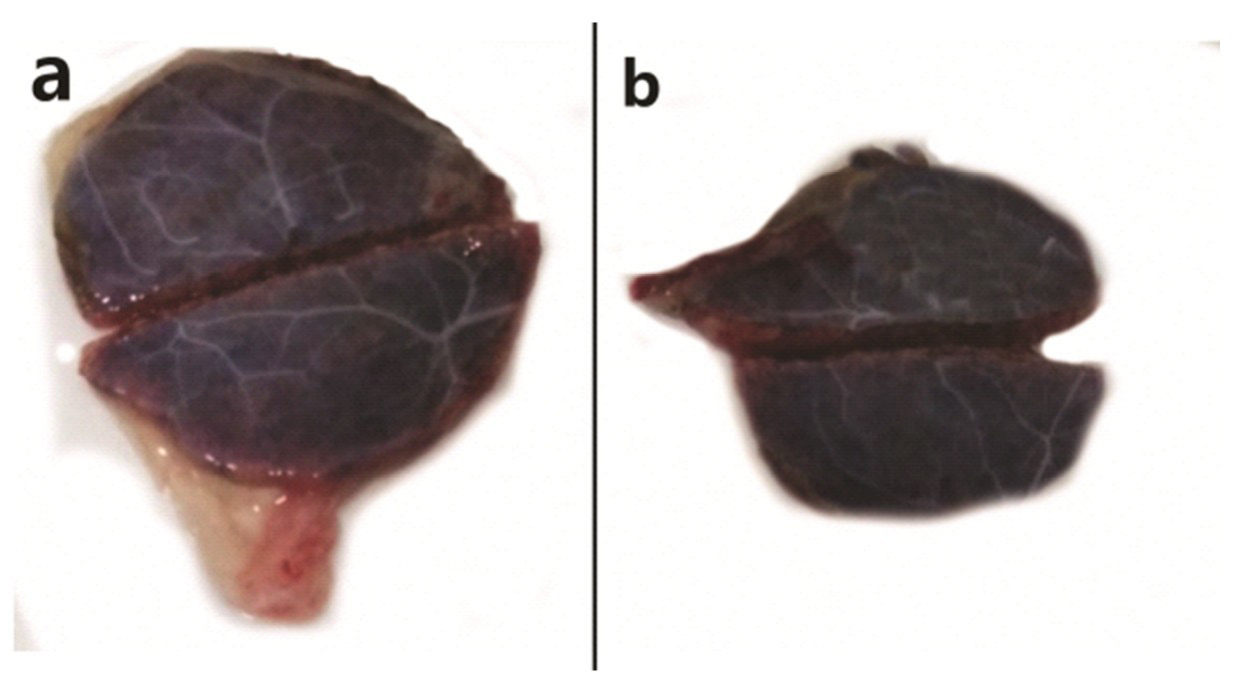
Photographic images depicting sheep placentome morphology. The photograph on the left depicts (a) healthy placentome collected from a control animal. The photograph on the right depicts (b) a range of reduction of placental volume, arterial and venous diameters abnormalities in placentome types collected from a stressed ewe.

Sections of all collected placental tissues were preserved in 10% buffered formalin according to routine protocols (Figure 3).

Following paraffin embedding and subsequently tissues were sectioned at 5 mm. Slides were stained with hematoxylin and eosin (H&E). All the staining techniques were performed as described by Bancroft and floyd (Bancroft and Floyd, 2013).

### Blood sampling

Five mL of blood was collected by jugular vein puncture from the experimental sheep, this blood sample was left in plain test tube at room temperature for 1 hr and then centrifuged for 10 min at 3000 rpm to obtain serum samples that were stored at −80°C for further analysis.

### Quantitative real-time PCR

Gene expression was quantified using the quantitative real-time PCR technique (qRT-PCR) for both more and less reactive sheep. All primers for genes encoding angiogenic protein genes (*NOS3, VEGF* and *HIF 1a*) were shown in Table 2 were designed using Primer-BLAST software (Sharawy et al., 2023). Trizol reagent was used to extract RNA from sections of collected placental tissues (Puregene, Genetix brands). The RNA pellet was dissolved fully after being eluted with 50 L of RNase-free water and incubated for 10 minutes at 55°C. The extracted RNA was reverse transcribed to cDNA in a 20 μl reaction using the SensiFASTcDNA synthesis method (Bioline, London, U.K.), in which 5 μl of RNA sample were mixed with 4 μl of 5x TransAmp Buffer, 1 μl of reverse transcriptase enzyme, and 10 μl of UltraPureDNase/RNase-free water. Thermal cycling was used to incubate the reaction mixture at 25°C for 10 min, 42°C for 15 min, and 85°C for 5 min. The cDNA samples were then diluted 1:10 in sterile DNase-free water and kept at -20°C for storage. 2 μl of cDNA template, 10 l SYBR Green PCR Master mix (SensiFAST SYBR NO- Imt kit, Bioline, London, UK), 0.8 μl of 10 μl forward and reverse primers (Vivantis Technologies Sdn Bhd., Malaysia), and 6.4 μl of sterilized Ultra-Pure DNase-free water were used to make a total volume of 20 u μl. The reaction mixtures were heated to 95°C for 10 minutes, then 40 cycles of 95°C for 15 seconds and 60°C for 1 minute, followed by 72°C for 15 seconds. Gel electrophoresis and melting curve analyses were used to determine the specificity of each primer. Furthermore, the effectiveness of each primer was estimated using the formula 'Efficiency = 1+10(-1/slope)'. The ^2−ΔΔCT^ approach published by Livak and Schmittgen (Livak and Schmittgen, 2001) was utilized to assess relative quantification of mRNA transcripts, with the β-actin gene serving as the housekeeping gene.

**Table 2:** Sequences of forward and reverse primers used for qPCR analyses.

**Gene**	**Forward primer (5′-3′)**	**Reverse primer (5′-3′)**	**Accession no**	**Product length**
*NOS3*	CAAGGTGACTTCCCAGAGACT	GAGAGAGGCGAGGGGAATCT	NM_001129901.1	173
*VEGF*	CTTGCCTTGCTGCTCTACCT	GCCCACAGGGATTTTCTTGC	AF071015.1	397
*HIF-1α*	ACAGCAAGAACCTGTTGCCT	TCAAGGCAGGGGAGCATACA	XM_027973633.2	151
β-actin	TGTTAGCTGCGTTACACCCT	GTCAAAGTCCTCGGCCACAT	NM_001009784.3	180

*NOS3* endothelial nitric acid synthase, *VEGF* vascular endothelial growth factor, *HIF* hypoxia inducing factor 1 alpha.

### Statistical analysis

Appropriateness of fit for the normal distribution of model-residuals of measured parameters (TAMV, PI, RI) and *NOS3, VEGF, HIF-1α* genes was visually assessed using normal probability plots (Q-Q-plots) and the Kolmogorov-Smirnov test. To determine the effect of time (wk) on TAMV, PI, RI on the expression of angiogenic proteins, a mixed model one-way analysis of variance (ANOVA), with time points as repeated measurements, was used. Post-hoc multiple pairwise comparisons were done according to a Tukey adjustment of error rate. Statistical analyses were conducted with commercial statistical software (SAS®, version 9.2, SAS Institute, Cary, NC). For all analyses, P ≤ 0.05 was defined as significant.

## Results

The pregnancies of the experimental sheep were 9 singletons, 11 twins. The confirmation of the pregnancy were observed at D 35- 40 of gestation. A mean total birth weight of 4.91 ± 0.91 kg and of 3.4 ± 0.54 kg was recognized in LR and MR pregnant ewes, respectively.

### Effect of maternal stress on uterine and umbilical blood perfusion and fetal biometry in pregnant ewes

#### Doppler and B-Mode measurements

Measurements of the uterine and umbilical arteries were successful in every attempt. Uterine artery: The uterine blood flow time average maximum velocity (TAMV) significantly increased in the LR than MR (P < 0.05). UtA-PI was higher during pregnancy in MR compared to LR ewes (Table 3). The increase in UtA-PI was significant at pregnancy wks 6 (P < 0.01), 10 (P > 0.05) and 12 (P < 0.01). Moreover, UtA-PI was tendentially higher at wk 14 (P = 0.054) (Table 3, Figure 1a). Furthermore, a higher UtA-RI was observed in MR ewes during the first 8 wks of pregnancy (P < 0.03) (Table 3, Figure 1a). Umbilical artery: UMA-RI was higher in MR versus LR animals, especially at weeks 14 (P < 0.0003) and 20 (P < 0.02) of pregnancy (Table 4, Figure 1b), while at the same time, a tendentially higher UMA-PI (P = 0.052, Table 4, Figure 1b) was observed. There was a significant increase of UMA-PI at wk 6, 8, 10 and 20 (P < 0.05 – 0.003). During the first 8 wks of pregnancy, the diameter of the amniotic vesicle was larger in LR than in MR animals (P < 0.03, Table 5, Figure 2a). Also, the fetal thoracic diameter (P < 0.002, Table 6, Figure 2b), as well as the fetal umbilical diameter (P = 0.05, Table 7, Figure 2c), was significantly larger in LR than MR pregnant ewes from wk 8-18. The fetal biometrical changes during pregnancy in all ewes, a linear increase (P < 0.0001) in fetal parameters, including thoracic diameter, umbilical cord diameter and fetal metacarpal length, occurred between wk 6 and wk 18 of gestation. The length of fetal metacarpal significantly increased in LR than MR fetuses (P < 0.05, Table 8, Figure 2d).

**Table 3:** Mean ± SD and ranges of the Blood flow timed average maximum velocity (UtA_TAMV: cm/s), Pulsatility index (PI) and Resistance index (RI) of uterine arteries ipsilateral to the pregnant horn between weeks 2 and 20 of pregnant Ossimi ewes (A). low letters (a, b) are different (p < 0.05).

**Time (wks)**	**TAMV-UTA-MR**	**TAMV-UTA-LR**	**PI- UTA-MR**	**PI- UTA-LR**	**RI- UTA-MR**	**RI- UTA-LR**
2	11.36±3.33 ^a^	9.27±1.75 ^b^	7.79±0.61 ^a^	5.19±0.6 ^b^	0.90±0.12 ^a^	0.77±0.08 ^b^
4	14.61±3.37 ^b^	19.77±1.10 ^a^	6.59±0.32 ^a^	3.40±0.8 ^b^	0.86±0.06 ^a^	0.70±0.06 ^b^
6	17.3±4.07 ^b^	26.95±3.40 ^a^	4.61±0.82 ^a^	3.39±0.88 ^b^	0.83±0.01 ^a^	0.70±0.05 ^b^
8	23.68±4.20 ^b^	31.44±3.31 ^a^	3.30±0.68 ^a^	2.28±0.80 ^b^	0.81±0.03 ^a^	0.81±0.04 ^b^
10	29.17±4.25 ^b^	37.8±2.01 ^a^	3.10±0.38 ^a^	1.45±0.39 ^b^	0.81±0.06 ^a^	0.64±0.07 ^b^
12	32.28±3.27 ^b^	40.22±1.73 ^a^	2.34±0.50 ^a^	1.31±0.17 ^b^	0.81±0.08 ^a^	0.66±0.03 ^b^
14	36.72±4.02 ^b^	46.12±2.40 ^a^	0.98±0.40 ^b^	0.91±0.19	0.72±0.05 ^a^	0.57±0.04 ^b^
16	42.63±6.09 ^b^	54.73±2.30 ^a^	0.95±0.27	0.97±0.24	0.77±0.02 ^a^	0.63±0.02 ^b^
18	43.91±5.24 ^b^	56.86±3.77 ^a^	1.23±0.10	0.98±0.15	0.77±0.02 ^a^	0.63±0.04 ^b^
20	48.61±4.38 ^b^	60.43±3.31 ^a^	2.65±0.17 ^a^	1.66±0.09 ^b^	1.00±0.01 ^a^	0.78±0.03 ^b^

Values are Mean ± SD of 20 (12 MR and 8 LR) ewes. Means with different superscripts (a,b) in rows are significantly different (P < 0.05).

**Table 4:** Mean ± SD and ranges of the Blood flow timed average maximum velocity (UtA_TAMV: cm/s), Pulsatility index (PI) and Resistance index (RI) of umbilical arteries between weeks 10 and 20 of pregnant Ossimi ewes (A). low letters (a, b) are different (p < 0.05).

**Time (wks)**	**TAMV-um-MR**	**TAMV-um-LR**	**PI-MR**	**PI-LR**	**RI-MR**	**RI-LR**
10	15.61±2.37 ^b^	22.34±2.18^a^	2.12±0.35	2.12±0.41	1.00±0.06	1.00±0.07
12	18.43±2.56 ^b^	24.91±2.27 ^a^	2.22±0.11^a^	1.42±0.29 ^b^	0.98±0.06 ^a^	0.78±0.04 ^b^
14	22.14±2.41 ^b^	27.18±3.17	2.68±0.14 ^a^	1.41±0.09 ^b^	0.96±0.02 ^a^	0.83±0.09 ^b^
16	22.85±2.41 ^b^	28.46±3.15^a^	1.54±0.08	1.49±0.05	0.82±0.4 ^a^	0.77±0.04 ^b^
18	28.03±2.61 ^b^	30.76±2.03 ^a^	1.12±0.11 ^b^	1.44±0.07 ^a^	0.97±0.11 ^a^	0.91±0.05 ^b^
20	27.80±4.94	25.94±5.46	1.59±0.39 ^b^	1.80±0.13 ^a^	0.92±0.13 ^b^	0.99±0.13 ^a^

Values are Mean ± SD of 20 (12 MR and 8 LR) ewes. Means with different superscripts (a,b) in rows are significantly different (P < 0.05).

**Table 5:** Amniotic vesicle diameter in cm (AV, mean ± SD) for less reactive (LR) and more reactive (MR) ewes during early pregnancy.

**Time (wks)**	**AV value (MR)**	**AV value (LR)**
**2**	1.55±0.12	1.6±0.16
**4**	2.25±0.32^b^	2.77±0.45^a^
**6**	4.15±1.6^b^	4.97±1.30^a^
**8**	5.73±1.12^b^	6.74±1.44^a^

Amniotic vesicle diameter (AV) with low letters (a, b) in rows are different (p < 0.05).

**Table 6:** Fetal thoracic diameter in cm (FTD) (FTD, mean ± SD) for less reactive (LR) and more reactive (MR) ewes during early pregnancy.

**Time (wks)**	**FTD values (MR)**	**FCHD values (LR)**
**6**	3.45±0.6	3.6±0.15
**8**	3.47±0.8^b^	3.9±0.22^a^
**10**	3.61±0.48^b^	4.5±0.33^a^
**12**	3.68±0.55^b^	4.61±0.31^a^
**14**	3.87±0.33^b^	4.67±0.23^a^
**16**	4.05±0.31^b^	4.78±0.8^a^
**18**	4.27±0.21^b^	4.82±0.9^a^

Fetal thoracic diameter (FTD) with low letters (a, b) in rows are different (p < 0.05).

**Table 7:** Umbilical cord diameter close to the fetal body in cm (UMD) (mean ± SD) for less reactive (LR) and more reactive (MR) ewes during early pregnancy.

**Time (wks)**	**UMD values (MR)**	**UMD values (LR)**
**6**	2.18±0.20^a^	1.85±0.04^b^
**8**	2.31±0.15 ^a^	1.93±0.08 ^b^
**10**	2.37±0.08	2.12±0.19
**12**	2.4±0.08	2.3±0.13
**14**	2.45±0.09	2.38±0.08
**16**	2.48±0.06	2.44±0.09
**18**	2.50±0.09	2.55±0.11

Umbilical cord diameter with low letters (a, b) in rows are different (p < 0.05).

**Table 8:** Metacarpal length (MCL) in cm (mean ± SD) for less reactive (LR) and more reactive (MR) ewes during early pregnancy.

**Time (wks)**	**MCL value (MR)**	**MCL value (LR)**
**6**	2.34±0.22	2.7±0.13
**8**	2.49±0.32 ^b^	2.73±0.22 ^a^
**10**	2.5±0.81 ^b^	3.04±0.15 ^a^
**12**	2.53±0.27 ^b^	3.24±0.31 ^a^
**14**	2.66±0.15 ^b^	3.26±0.34 ^a^
**16**	2.73±0.4 ^b^	3.36±0.30 ^a^
**18**	2.75±0.33 ^b^	3.4±0.31 ^a^

Metacarpal length with low letters (a, b) in rows are different (p < 0.05).

### Effect of maternal reactivity/anxiety on the gestation period, birth weight and fetal numbers

The gestation period was significantly lower in MR animals than in LR ones (MR sheep: 148.2 ± 1.49 d; LR sheep: 153.7 ± 1.54; (P < 0.05, Table 9). Fetal numbers were 1.57 ± 0.65 (MR sheep) in MR vs. 1.65 ± 0.50 (LR sheep) respectively. Furthermore, the total birth weight was higher in LR sheep than in MR sheep (3.85 ± 1.35 kg vs. 3.64 ± 1.24 kg; P > 0.05, Table 8).

**Table 9:** Effect of maternal reactivity/anxiety on the gestation period (days), birth weight (kg) and fetal numbers.

**items**	**More reactive (MR)**	**Less reactive (LR)**	***P*-value**
Gestational length	148.2 ± 1.49^b^	153.7 ± 1.54^a^	P > 0.05
Fetal weight	3.64 ± 1.24 ^b^	3.85 ± 1.35 ^a^	P > 0.05
Fetal number	1.57 ± 0.65 ^b^	1.65 ± 0.50 ^a^	P > 0.05

Variables with different letters (a,b) in the same row are significantly different at*P*< 0.05.

### Hematoxylin and eosin-stained sections and Morphometric analysis

**Figure 4:**
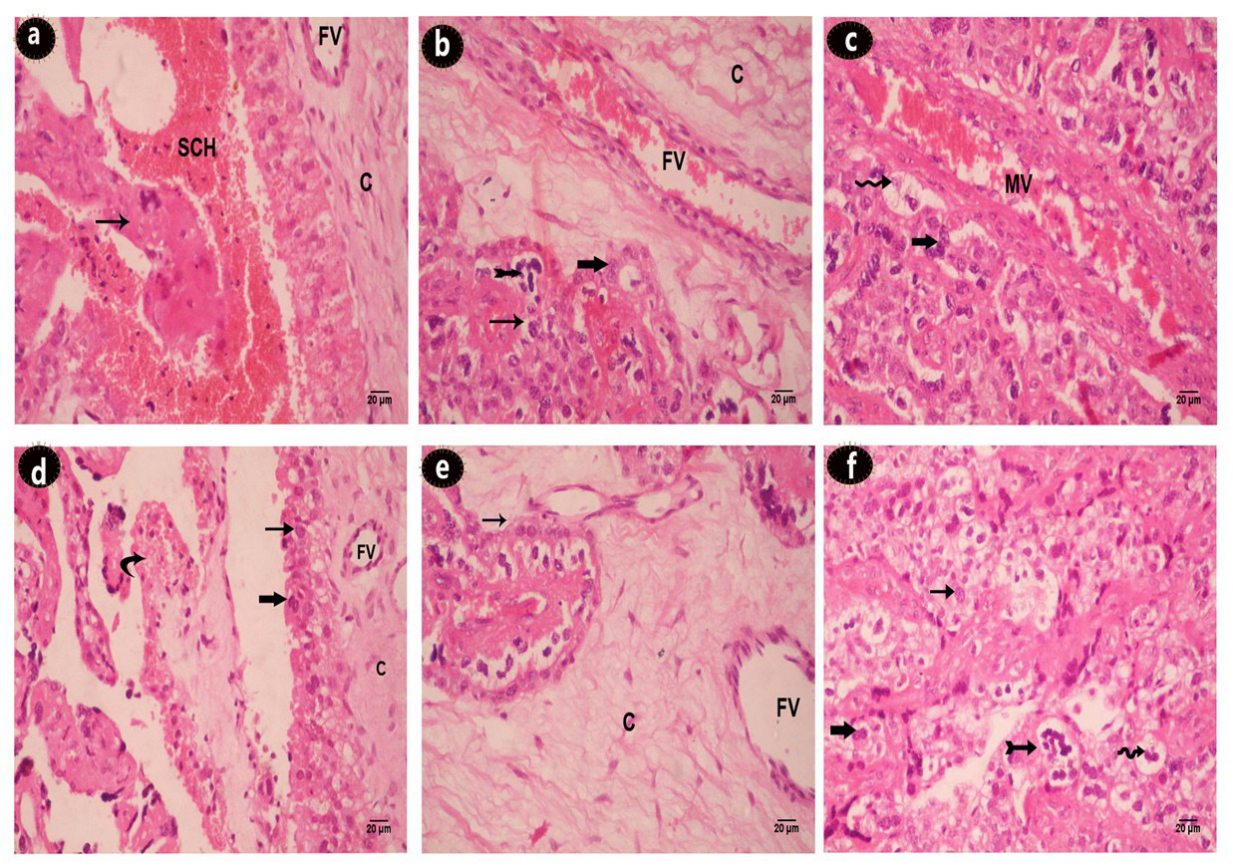
Placentome histology of (a,b,c) normal placentome from a control ewe and (d,e,f) placentome from stress sheep (H&E). (a) subchorial hematoma (SCH); normal chorium (C); fetal blood vessels (FV); villi (thin arrow); (b) binucleated trophoblast (thick arrow); giant cell (pointed arrow); mononucleated trophoblast (thin arrow); (c) maternal blood vessels (MV); vacuolated cell (wavy arrow); bi-nucleated trophoblast (thick arrow); (d) villous hypovascularity (curved arrow); trophoblastic hyperplasia (thick arrow); (e) chorial fibrosis (c); abnormal fetal blood vessels (FV); (f) vacculation (wavy arrow); syncytial knotting (pointed arrow); villous necrosis (thin arrow).

The histology of placental tissues either from MR or LR ewes were assessed in light microscopy of HE-stained slides. Placentome of a healthy control ewe show the subchorial haemophagus area where differences were observed with amount of erythrocyte debris, with clear corium and intact trophoblasta layer (Figure 3a and b). Analysis of the placental tissue showed that stress (maternal temperament) caused severely compromised placental morphology. These changes include syncytial knotting, villous hypovascularity, villous fibrinoid necrosis, reduction in number of villous trees. The thickening of trophoblastic basement membrane, trophoblastic hyperplasia, perivillous fibrin deposition, chorial fibrosis (Figure 4a, b, c, d, e and f).

### Effects of maternal temperament on the relative expression of angiogenic genes

The effects of maternal temperament on the expressions of genes related to angiogenic proteins in the placental tissues were evaluated using qRT-PCR. The expressions of angiogenic protein genes (*NOS3, VEGF*) and *HIF-1a* were decreased significantly in the current study in ewes showed anxiety temperament than the calm ones (P < 0.05, Table 10).

**Table 10:** The expressions of *NOS3*, *VEGF* and *HIF-1α* genes in blood of more and less reactive pregnant Ossimi ewes.

**Gene**	**LR**	**MR**	***P*-value**
*NOS3*	1.00 ± 0.00	0.3 ± 0.0.13 ∗∗	0.03
*VEGF*	1.00 ± 0.00	0.19 ± 0.09**∗∗**	0.0001
*HIF-1α*	1.00 ± 0.00	0.25± 0.41**∗∗**	0.001

∗∗ Variables with different superscript in the same row are significantly different at P < 0.05.

## Discussion

The purpose of this study was to see how the temperament of the dam affected foetal growth as well as maternal and umbilical Doppler parameters in sheep during pregnancy. It has been reported that non-invasive B-mode and Doppler ultrasound can be used to track intrauterine foetal development and/or uterine blood flow changes in pregnant animals (Meler et al., 2021; Muniz et al., 2022; Wu et al., 2021). Maternal anxiety has been shown to have an effect on Doppler and fetometric parameters in some studies (Bayrak and Sancak, 2021). The current study found that UtA-PI in ewes was higher in MR than LR animals between weeks 8 and 20 of pregnancy. Similar results were obtained in humans. Increasing in UtA-RI and UTA-PI in anxious pregnant animals before week 21was investigated (Vythilingum et al., 2010). However, in the same study, anxiety was not related to UtA-RI at week 22 of pregnancy. On the other hand, found no correlation between UTA-RI and UMA-RI and maternal distress (Mendelson et al., 2011).

The placenta in sheep is made up of several hundred placentomes and is of the cotyledonous type (Brzozowska et al., 2020). Every placentome is a functional unit made up of the fetal part (cotyledon), which is created by the union of the vascularized allantois and the avascular chorion, and the maternal part (caruncle), which is formed from the endometrium covering the uterine papillae (Sammin et al., 2009). Maternal vascularization is generated by branches of uterine radial arteries that enter the caruncle from its base and then extend over its convex surface, according to research on the architecture of the placenta in sheep.

The umbilical vascular system in sheep is composed of two veins and two arteries. These vessels do not split along the umbilical cord; instead, they split into two umbilical trunks, each comprising an artery and a vein, in the chorioallantoic region. Research on sheep membranes using postpartum morphometric analysis has extensively documented this behavior. The Doppler indices derived from previous investigations have yielded valuable clinical insights. On the other hand, not much information is available to describe blood flow in the blood vessels that create the placentomes' direct vascularization. Doppler spectrum could be a valuable factor to take into account when determining if the fetus is developing normally (Acharya et al., 2004).

The effect of maternal temperament on blood flow of dam and fetus in goats were studied by Samir et al., (Tomasz et al., 2019) using Doppler indices of blood and placental structure, they found that sedation and relaxation of pregnant dams have significant effects on estimated Doppler parameters as relaxation increase blood flow. Investigation of placental structural variations delivers understanding of uterine and placental arterial blood drift, ripening of placental principally in the final stages of gestation (Tomasz et al., 2019).

The increased Doppler indices observed in this study could be attributed to a number of factors discussed in the international literature. Stress hormone levels (Teixeira et al., 2005) and transient changes in maternal hormone concentrations influence uterine blood flow parameters. The current study found that PI and RI levels were higher in MR pregnant sheep than in LR pregnant sheep (Teixeira et al., 1999). Those findings could be attributed to changes in cortisol levels, which have been discussed in particular in women with high anxiety levels (Teixeira et al., 2005). Other data on changes in uterine and umbilical blood flow velocity, particularly blood flow impedance indices, support the current study's findings (Zheng and Hu, 1995; Sağol et al., 2002). Furthermore, decreased RI and PI values in the uterine and umbilical arteries were linked to higher blood flow volume, which was attributed to uterine vascular vasodilation in combination with placental derived growth factors and local estrogen (and other steroids) synthesis from the ewes' placenta (Magness and Rosenfeld, 1989)-(Rosenfeld et al., 2001).

Many factors influence intrauterine fetal growth, including maternal, fetal, placental, and genetically determined growth potential (Chard et al., 1992; Cowan et al., 2021; Gardosi, 2012; Steer, 1992; Vieira et al., 2019). LR sheep had a larger amniotic vesicle diameter than MR sheep during the first 8 weeks of pregnancy. Later, from wk 8 to the end of pregnancy, the fetal thoracic and umbilical cord diameters were significantly larger in LR ewes. Similar results were reported in pregnant goats, the placentome and fetal thoracic diameter, as well as the fetal metacarpal length, were significantly larger in LR than in MR animals (Rosenfeld et al., 2001).

In addition, there was a significantly higher FCD and UMD. Similar findings in humans have been reported in terms of head circumference, width, and femur length (Shafizadeh and Mehdizadeh, 2008). In this study, the total birth weight of lambs was significantly higher in LR than in MR sheep. These findings were similar to those of Field and Diego (2008) and Henrichs et al. (2010) in maternally distressed humans and ewes following artificial intrauterine growth restriction (Gahlawat et al., 2021; Lazarou et al., 2021; Liao et al., 2021). In contrast, others found that stressed lambs had a higher birth weight (Macías-Cruz et al., 2021; Meza-Herrera et al., 2015; Rakers et al., 2013).

The more and less reactive sheep have different gestation durations. Smith et al. (Smith et al., 2008) found that sheep exposed to stress within the first 6 days after conception had shorter gestation durations than control ewes.

Fetal counts were lower in MR than LR experimental lambs. This could be due to LR animals having a higher ovulation rate than MR animals (Hart et al., 2006). The gestation lengths of MR and LR animals were clearly different. When ewes were stressed within the first 6 days following conception, Smith et al. (2018) found that they had shorter gestation lengths than control ewes. According to the findings of the B-mode and Doppler investigations, alterations in uterine and/or umbilical blood flow may have a significant influence on intrauterine fetal growth. Non-invasive imaging of changes can be used to demonstrate the effects of animal temperament. Another findings are controversial to ours, they noticed that no obvious alterations in the fetal aortic blood flow among nervous and calm groups. And no effect of maternal anxiety on the fetal circulation in the course of pregnancy (Hart et al., 2006).

The circulatory system guarantees that oxygen and nutrients are delivered to all cells, tissues, and organs. Cells can endure prolonged stress by activating a number of genes involved in angiogenesis, glucose metabolism, and cell proliferation via transcriptional activation. The oxygen-sensitive transcriptional activator HIF-1 (hypoxia-inducible factor-1) is an important transcriptional modulator of hypoxic response. HIF-1 was discovered to be a master regulator of angiogenesis (Zimna and Kurpisz, 2015).

The effects of maternal anxiety on the expression of angiogenic proteins were assessed in the pregnant sheep for the first time in the present study. Maternal anxiety decreased the expression of angiogenic proteins at parturition in reactive sheep more than in normal sheep. The variation in the expressions of *NOS3, VEGF* and *HIF-1α* protein genes may explain the variation in both Doppler and fetal biometry parameters in the present study (Guo et al., 2022; Kim et al., 2002; Miyashita-Ishiwata et al., 2022). Other studies have proven that the expression of *VEGF* protein gene is upregulated by *HIF-1α* protein gene. The decrease in the expressions of angiogenic proteins may be related to other factors that decrease the diameter of the maternal blood vessels. In conclusion, this study reports the effects of maternal stress on uterine blood flow, fetal heart rate, length of pregnancy, and fetal birth weight in sheep. Maternal stress has a significant effect on uterine artery blood flow especially in the second half of pregnancy. Maternal stress shortens the length of pregnancy and fetal birth weight but not the fetal heart rate during pregnancy.

## Conclusions

The current study proposed that maternal temperament of sheep has a very important effect on various parameters of fetus and uterus during the gestation period of pregnant Ossimi sheep including: blood circulation of dams and their feti, expressions of angiogenic proteins, measurements of amniotic vesicles, fetal thoracic diameters and mercaptal length. And Doppler sonography of uterine arteries and amniotic vesicles revealed that the UtA-PI was upserged in MR compared to LR ewes and UMA-RI was higher in fetuses of MR than LR ewes. In addition, histological examination of placental tissues depicted numerous distorted placental morphology. Blood samples were taken to extract RNA and perform real time PCR which revealed that mRNA expressions of angiogenic protein genes as NOS3, VEGF and HIF-1α were significantly diminished as a response to maternal temperament. These findings recommend that all the obtained alternations can be used as a tool to monitor the health state and the biometry of pregnant Ossimi ewes which can improve sheep productivity and so sheep breeders have the ability to avoid the adverse effects of maternal temperament on both ewes and their feti.
